# That imaging is necessary to avoid missed diagnoses even in pregnant women: Case report

**DOI:** 10.1097/MD.0000000000042636

**Published:** 2025-08-08

**Authors:** Peng Guo, Wulan Cao, Lilan Wang

**Affiliations:** a Department of Obstetrics and Gynecology, Zhongshan City People’s Hospital, Zhongshan, Guangdong, China.

**Keywords:** ECMO, fetal death, lung cancer, postpartum hemorrhage, pregnancy

## Abstract

**Rationale:**

The clinical symptoms and signs of lung cancer patients during pregnancy are not specific, making early clinical diagnosis difficult. The unique physiological and pathological changes in pregnant women significantly increase the risk of pulmonary embolism during pregnancy, but its diagnosis is more challenging compared to nonpregnant patients.

**Patient concerns:**

This article reports a case of a pregnant woman with lung cancer misdiagnosed as pulmonary embolism, ultimately leading to severe conditions requiring extracorporeal membrane oxygenation (ECMO) treatment. During ECMO support, the patient developed spontaneous intrauterine fetal death and natural expulsion.

**Diagnoses:**

The final pathological diagnosis is lung adenocarcinoma.

**Interventions:**

After admission, the patient received ECMO support, anti-infection, anticoagulation, blood transfusion, liver protection, and nutritional support. During the fetal expulsion period, blood transfusion was administered to improve coagulation, and medications were actively used along with the placement of an intrauterine balloon to promote uterine contraction and hemostasis. After a diagnosis of lung cancer, targeted therapy with alectinib was administered.

**Outcomes:**

The patient avoided postpartum hemorrhage, safely passed the critical period, and the antitumor treatment for lung cancer was effective, leading to a favorable prognosis.

**Lessons:**

This case reminds us that pregnant patients with recurrent respiratory symptoms should undergo necessary imaging examinations promptly to avoid misdiagnosis. Pregnant patients receiving ECMO therapy are at risk of sudden fetal death and spontaneous fetal expulsion, with a significantly increased risk of severe hemorrhage during the expulsion period. It is crucial to detect and prevent hemorrhage in a timely manner during the expulsion period to avoid serious complications.

## 1. Introduction

Clinical manifestations of pulmonary diseases during pregnancy can easily be confused with the physiological state of pregnancy. Additionally, the rapid progression of diseases during pregnancy and concerns about the impact of imaging examinations on the fetus often lead to delays in diagnosis and treatment. Serious pulmonary diseases during pregnancy include lung cancer and pulmonary embolism (PE). The incidence of cancer during pregnancy is approximately 0.1%, accounting for about 0.07% to 0.1% of all malignant tumors, with lung cancer during pregnancy being extremely rare. However, with the increase in the average age of pregnancy and the number of smoking women, the incidence of lung cancer during pregnancy has been rising over the past 2 decades.^[1,2]^ Pregnancy with lung cancer is a special disease with a poor prognosis, and early diagnosis can help improve maternal and fetal outcomes. The incidence of PE during pregnancy is approximately 0.1%, with a mortality rate of 3%, significantly higher than in nonpregnant patients.^[3]^ Therefore, once a suspected case of PE is identified, early diagnosis and treatment are essential, along with the formulation of specialized perinatal management plans. We report the changes and management of a second trimester patient with suspected PE who was eventually diagnosed with lung cancer after veno-arterial extracorporeal membrane oxygenation (VA-ECMO) treatment.

## 2. Case description

The patient is a 29-year-old female with no previous medical history, no smoking history, and no family history of tumors. She has not experienced symptoms such as fever, hemoptysis, sputum production, or chest pain. The patient presented to an external hospital on January 15, 2024, with a 1-month history of dyspnea and mild cough at 24 weeks + 3 days of gestation.

Testing for novel coronavirus, influenza virus, and mycoplasma pneumoniae was negative, and there were no significant abnormalities in infection markers. Coagulation function tests indicated D-dimer levels of 20 mg/L and fibrinogen levels of 1.5 g/L, initially suggesting PE. However, due to pregnancy, computed tomography pulmonary angiography (CTPA) was not performed. After treatment with low-molecular-weight heparin for anticoagulation, her cough slightly improved, and she was discharged at her own request. After discharge, she continued to experience shortness of breath.

**Figure 1.**
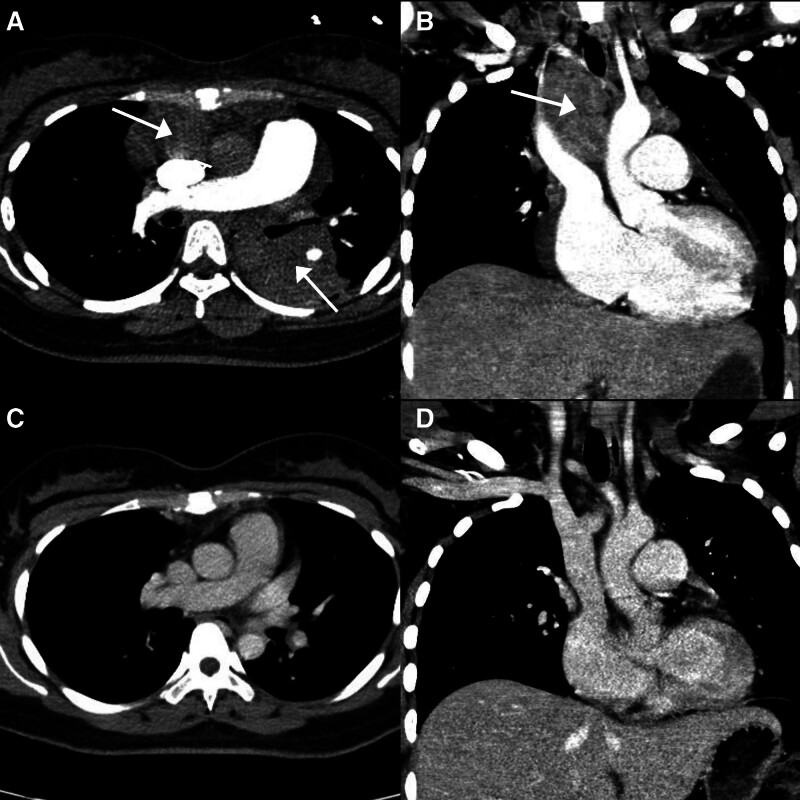
CTPA on January 25, 2024 (A, B) and repeat chest CT on December 19, 2019 (C, D). (A) Transverse image shows left lung mass and multiple mediastinal nodules. (B) Coronal image shows multiple mediastinal nodules. (C, D) CT shows that left lung mass had almost disappeared and multiple enlarged lymph nodes in mediastinum had significantly reduced. CT = computed tomography, CTPA = computed tomography pulmonary angiography.

The patient presented to our emergency department at 23:30 on January 24, 2024, at 25 weeks and 5 days of gestation, with worsening dyspnea and chest pain for 1 day. Physical examination revealed normal blood pressure, tachypnea (30 breaths/min), and peripheral oxygen saturation of 80%. Arterial blood gas analysis showed hypoxemia (PaO₂ 71 mm Hg) and lactic acidosis (lactate 4.3 mmol/L; see details in Supplement 1, Supplemental Digital Content, https://links.lww.com/MD/P60 for full laboratory data). No significant improvement was observed after high-flow oxygen therapy. On January 25, 2024, at 4:00, the patient’s shortness of breath worsened further, accompanied by a decrease in blood oxygen and blood pressure (saturation of pulse oxygen dropped to 70%, blood pressure dropped to 56/32 mm Hg), and noninvasive ventilation (NIV) did not improve the condition. A cardiac ultrasound indicated an enlarged right heart, severe pulmonary hypertension, and severe tricuspid regurgitation (see detail in Supplement 2, Supplemental Digital Content, https://links.lww.com/MD/P61), with a D-dimer level of 2.19 mg/L, initially suggesting obstructive shock caused by PE. At 05:20, VA-ECMO support was initiated, and by 06:05, the patient was successfully stabilized, with blood pressure and oxygen saturation restored. The anticoagulation regimen was unfractionated heparin (UFH). She was then admitted to the intensive care unit for further monitoring and treatment. NIV was continuously administered (Mode: CPAP, FIO_2_ 100%, VT 483 mL, f 38 breaths/min, PEEP 5 cm H_2_O). CTPA revealed no pulmonary embolism, but multiple nodules and masses were found in the bilateral supraclavicular fossa and mediastinum, some of which were fused into clusters (the largest located in the left posterior mediastinum, extending into the left lung, approximately 43 mm × 60 mm in size; Fig. 1A, B). Lymphoma was highly suspected through CTPA, with the differential diagnosis including a primary lung tumor with lymph node metastasis.

**Figure 2.**
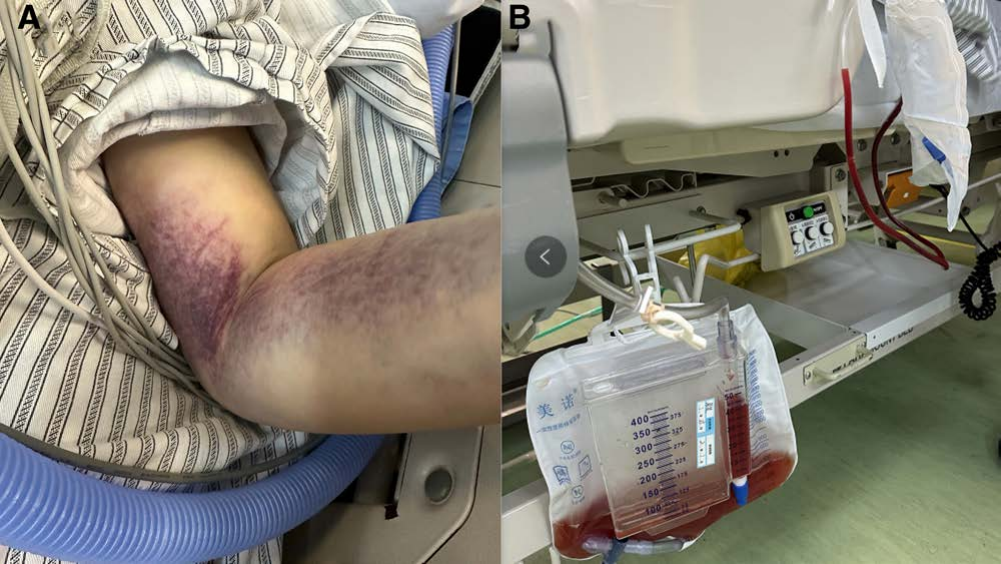
DIC state of the patient: ecchymoses (A) and hematuria (B). DIC = disseminated intravascular coagulation.

Relevant examination was performed after admission. Laboratory tests showed significantly increased infection indicators, moderate anemia, significant thrombocytopenia and coagulopathy, abnormal liver function and heart function (see detail in Supplement 1, Supplemental Digital Content, https://links.lww.com/MD/P60). Blood gas analysis indicated significant metabolic acidosis. The patient’s shortness of breath was considered related to metabolic acidosis and tumor compression of the airway. Pulmonary hypertension and right heart enlargement were considered secondary changes. The patient developed gross hematuria and extensive ecchymoses following ECMO therapy, suggesting disseminated intravascular coagulation (DIC; Fig. 2A, B). After admission, the patient continued to receive ECMO support, anti-infection, anticoagulation, blood transfusion to improve coagulation, liver protection, and nutritional support treatments. A multidisciplinary consultation was held, and on January 26, 2024, an ultrasound-guided left cervical lymph node biopsy was performed, with pathological results indicating a few suspicious tumor cells.

The emergency ECMO procedure was performed with normal fetal heart monitoring, but subsequent bedside ultrasound after admission revealed intrauterine fetal demise. On January 27, 2024, at 20:50, the patient began to experience regular lower abdominal pain. As she was at high risk for postpartum hemorrhage, large amounts of cryoprecipitate (10 units), fibrinogen (4 g), fresh frozen plasma (400 mL), platelet (2 units), and red blood cells (2 units) were administered before fetal expulsion to improve coagulation and prevent postpartum hemorrhage. At 22:20 on January 27, 2024, the patient naturally expelled a stillborn male fetus weighing 1100 grams, with no visible abnormalities. The placenta and membranes were basically intact, with first-degree perineal tear and minimal vaginal bleeding. After fetal expulsion, oxytocin (20 U), tranexamic acid (1 g), carbetocin (100 µg), and calcium gluconate (1 g) were administered intravenously to enhance uterine contraction and hemostasis, and a uterine hemostatic balloon was placed with the consent of the patient and her family. Anticoagulation therapy was stopped after the onset of labor signs. After fetal expulsion, no obvious signs of active bleeding were observed. After consultation between the obstetric team and ECMO team, anticoagulation was restarted 12 hours after fetal expulsion, and gradually increased based on the patient’s indicators, circulation, and hemoglobin stability. No significant active bleeding was observed 24 hours after fetal expulsion, and the uterine hemostatic balloon was successfully removed. Ultrasound reexamination of the uterus and adnexa 4 days after fetal expulsion showed no significant abnormalities.

On January 27, 2024, an ultrasound-guided mediastinal mass biopsy was performed again, with pathology indicating metastatic cancer. Immunohistochemical characteristics supported a diagnosis of metastatic lung adenocarcinoma. On February 1, 2024, the patient sucked out a large amount of bloody fluid through the mouth, necessitating tracheal intubation to protect the airway. Bronchoscopy revealed active bleeding in the nasal cavity, which was controlled with nasal packing and blood transfusion to improve coagulation. Genetic testing for lung cancer revealed an ALK fusion mutation. With the consent of the family, the patient began targeted therapy with alectinib 600 mg bid on February 1, 2024. Follow-up cardiac ultrasound showed a decrease in pulmonary pressure. ECMO was successfully weaned off on February 6, 2024, and tracheal intubation was removed on February 8, 2024. Vital signs were stable, and she was transferred to the general respiratory ward on February 9, 2024. In the general ward, the patient continued antitumor treatment, gradually improved, and was discharged on February 15, 2024. At discharge, her activity level was normal, with no shortness of breath or chest tightness.

**Figure 3.**
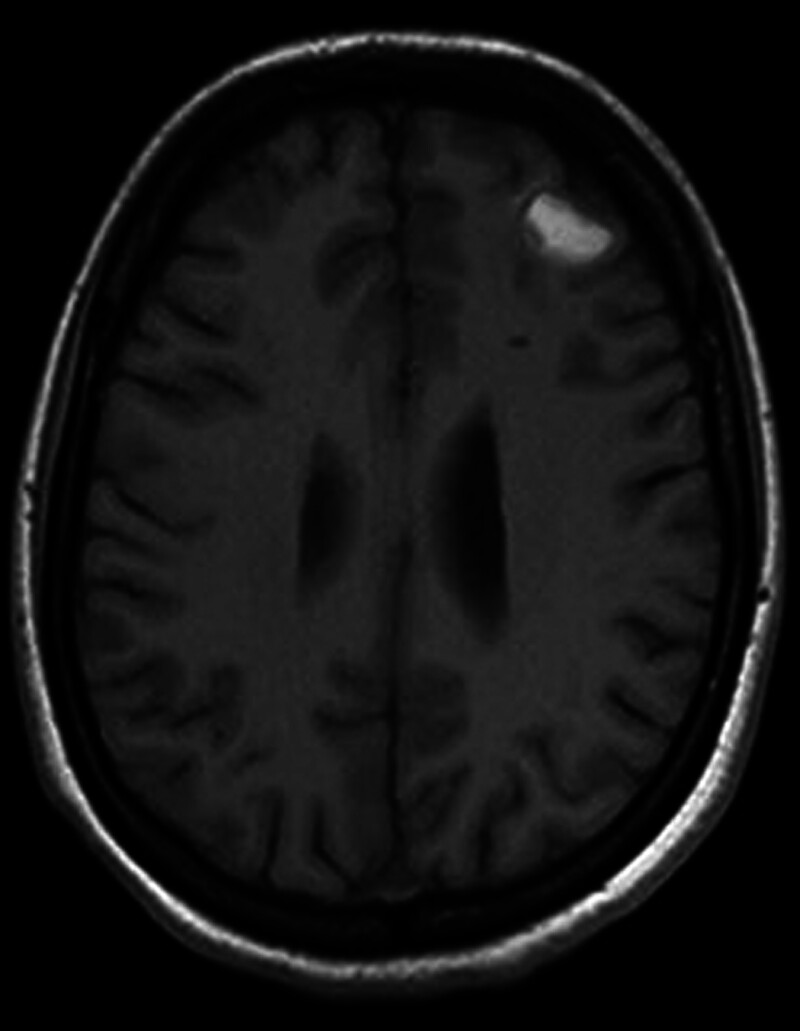
MRI indicates intracranial hemorrhage. MRI = magnetic resonance imaging.

From February 24 to February 28, 2024, the patient was readmitted to the neurosurgery department due to a small amount of intracranial hemorrhage (subacute) and improved with conservative treatment before being discharged (Fig. 3). Follow-up chest CT on February 26, 2024, indicated that the original left lower lung hilar mass had almost disappeared, with only patchy ground-glass opacities remaining (Fig. 2C). The multiple enlarged lymph nodes in the bilateral supraclavicular fossa and mediastinum had significantly reduced (Fig. 2D). On March 19, 2024, a follow-up ultrasound of the uterus and adnexa revealed a corpus luteum cyst, indicating the patient’s recovery of ovulation.

## 3. Discussion

### 3.1. Early detection and diagnosis of severe pulmonary diseases during pregnancy

Lung cancer during pregnancy is extremely rare, but the number of cases has been increasing in recent years due to smoking in young women and delayed childbearing. Because its clinical characteristics are atypical and easily confused with early pregnancy symptoms, diagnosis is difficult, often being identified in late pregnancy with poor prognosis. Reports indicate that 92% of pregnant lung cancer patients are diagnosed at an advanced clinical stage, with rapid disease progression and a 1-year survival rate of only 52.5%.^[4]^ Misdiagnosis, missed diagnosis, and poor prognosis can be attributed to several factors: Pregnancy may mask cancer symptoms, as clinical manifestations related to lung cancer, such as general fatigue, cough, chest tightness, and dyspnea, are often attributed to physiological changes during pregnancy.^[5]^ Due to concerns about radiation, pregnant women are less likely to undergo radiological examinations, even if recommended by physicians. Increased hormone levels during pregnancy and decreased maternal immune function may accelerate tumor growth.^[6,7]^

The most common histological type of lung cancer during pregnancy is non-small cell lung cancer, with lung adenocarcinoma being the most prevalent subtype, accounting for approximately 80%.^[8]^ Delayed pregnancy and a history of smoking have been identified as potential risk factors for lung cancer during pregnancy.^[2]^ Some scholars suggest that pregnant women over 30 years old who smoke should be evaluated for potential lung cancer if they develop respiratory symptoms during pregnancy.^[9]^ This case is particularly unusual as the patient was a young, nonsmoking woman without any traditional risk factors for lung cancer. However, suspected lung cancer in pregnant women should be promptly evaluated with appropriate examinations to achieve early diagnosis. Tumor marker levels are related to the histological type and clinical stage of the tumor and can assist in diagnosing lung cancer during pregnancy.^[10]^ However, most serum biomarkers are generally ineffective for early diagnosis or screening of tumors. If malignancy is highly suspected, the necessity of examinations should be emphasized to the patient and her family, and radiological evaluations should be conducted with informed consent to avoid missed diagnoses. The significant risk threshold for fetal damage is set at 100 mGy.^[11]^ The radiation doses from chest X-rays, CT, and MRI during pregnancy are all below 100 mGy (10 rad), so these can be used with sufficient abdominal shielding.^[12]^ Ionizing imaging procedures should be avoided as radiation may affect fetal survival and development.^[13]^ PET and bone scans are not recommended for evaluating distant metastases due to the risk of fetal malformations secondary to radioactive substances. Painful skeletal sites can be screened with X-rays to exclude bone metastasis. The definitive diagnosis of lung cancer is made through tissue biopsy, such as CT/ultrasound-guided needle biopsy, bronchoscopy, or superficial lymph node biopsy, and in the late stages of pregnancy, this can be done in a well-equipped hospital with adequate fetal monitoring.^[14]^

The incidence of PE during pregnancy and the puerperium is low, with atypical clinical symptoms and insidious onset. It is one of the leading causes of maternal mortality and represents a significant potential risk to obstetric quality and safety. For suspected cases, a comprehensive evaluation incorporating clinical manifestations and auxiliary examinations should be performed to achieve an early and accurate diagnosis, in order to facilitate the timely initiation of personalized and standardized treatment. For pregnant patients suspected of PE, especially those with symptoms of deep vein thrombosis, compression ultrasound (CUS) should be performed. If deep vein thrombosis is positive, treatment for venous thromboembolism should be initiated immediately. If CUS is negative and PE is still highly suspected clinically, further radiological imaging should be performed. CTPA and ventilation/perfusion scanning are highly significant for diagnosing PE during pregnancy. The radiation dose to the fetus is far below the threshold for fetal-related complications (50–100 mSv).^[15]^ CTPA provides clear visualization from the main pulmonary artery to subsegmental branches and has high diagnostic accuracy in clinical practice, making it the gold standard for diagnosing PE. In this case, PE was suspected, but there was no significant improvement with standard anticoagulant therapy. Additionally, the initial finding of decreased fibrinogen was inconsistent with pregnancy-related PE. At this point, the accuracy of the PE diagnosis should be highly questioned, and timely communication for radiological imaging examination should be undertaken to confirm the diagnosis, thereby avoiding delays in treatment.

### 3.2. Management of hemorrhage prevention during fetal expulsion in special patients

The main causes of hemorrhage during mid-pregnancy fetal expulsion include retained placenta, uterine atony, coagulation disorders, obstetric injuries, and psychological factors. During the process of fetal expulsion, it is crucial to closely monitor the patient’s condition, promptly identify the causes of bleeding, and administer appropriate treatment. Early identification of high-risk groups for postpartum hemorrhage (PPH) and predicting its occurrence prenatally can guide clinicians in implementing effective interventions at the earliest opportunity. This is essential for reducing the incidence of PPH and improving adverse outcomes.

The most commonly used anticoagulant during ECMO is UFH, favored for its rapid onset, easy reversibility, and established monitoring protocols.^[16]^ However, this anticoagulation strategy carries risks of heparin-induced thrombocytopenia and significant bleeding. This case was highly suspected of heparin-induced thrombocytopenia due to the markedly low platelet level after ECMO. Low-molecular-weight heparin may be considered as an alternative due to its little effect on platelets and sufficient pharmacokinetic data. This mode of anticoagulation during ECMO, although its use is limited by challenges in dose adjustment and reversal, has been reported to be feasible and safe in observational trials and case series.^[17,18]^

Bleeding is the most common complication associated with ECMO (32%). It is recommended to use uterotonic drugs promptly during the perinatal period and to proactively prevent PPH with measures such as prophylactic use of the Bakri balloon.^[19,20]^ PPH and DIC are often mutually reinforcing, with postpartum hemorrhage potentially triggering DIC, and coagulation disorders of DIC exacerbating postpartum hemorrhage.^[21]^ The patient is in a DIC state following ECMO, placing her in a high-risk category for postpartum hemorrhage. Therefore, proactive management is essential to prevent PPH and avoid further deterioration of her condition. Supplementing relevant blood products is the most important means of correcting coagulation dysfunction. For severe DIC patients, following a massive transfusion protocol is recommended to correct anemia and coagulation abnormalities.^[22]^

A retrospective international multicenter study has shown that among pregnant women receiving ECMO treatment for acute respiratory or cardiac diseases during pregnancy, the fetal mortality rate is 47%.^[23]^ Another retrospective multicenter cohort study also indicated that 36% of pregnant women experienced fetal death and natural expulsion following the use of ECMO.^[24]^ In this patient, spontaneous fetal death and uterine contractions leading to natural fetal expulsion occurred after ECMO, with the placenta and membranes being basically intact. The primary factors for hemorrhage were coagulation disorders and uterine atony. Therefore, anticoagulants were discontinued upon the onset of labor signs, and large amounts of plasma, platelets, and fibrinogen were transfused before fetal expulsion to improve the DIC condition. After fetal expulsion, tranexamic acid, calcium supplement, and uterotonic agents were actively used, along with intrauterine balloon tamponade to promote uterine contraction and hemostasis. Study shows that tranexamic acid can reduce mortality due to postpartum hemorrhage without adverse effects and should be administered as soon as possible after bleeding begins.^[25]^ Intrauterine balloon tamponade achieves hemostasis by applying physical pressure to the uterine wall, promoting uterine contraction. This method is widely used domestically and internationally with good clinical outcomes.^[26]^ Studies have shown that performing intrauterine balloon tamponade in cases of less blood loss reduce the volume of blood loss, decrease the transfusion rate and severity of complications, and lower the risk of hysterectomy.^[27]^ Given the specific circumstances of this case, the patient could not be moved. Thus, an experienced obstetrician placed the intrauterine balloon tamponade without moving the patient’s ECMO cannulated lower limb. With active preparation for fetal expulsion and preventive measures for postpartum hemorrhage, this case avoided severe postpartum hemorrhage and surgical intervention, successfully navigating the critical period.

## 4. Conclusion

For pregnant patients with recurrent respiratory symptoms, necessary imaging examinations should be promptly performed to achieve a rapid diagnosis. The management of the fetal expulsion period in mid-pregnancy is crucial, especially for special or high-risk patients. Active preparation and preventive measures can avoid severe postpartum hemorrhage and surgical interventions, potentially saving the patient’s life. Pregnant patients on ECMO represent a uniquely high-risk population, in whom spontaneous intrauterine fetal death and natural labor for fetal expulsion may occur at any time during therapy. It is essential to promptly identify and prepare for the prevention of hemorrhage during fetal expulsion to avoid severe complications. Certainly, this represents only a single case of diagnostic and therapeutic experience, which has inherent limitations. To further improve outcomes for both pregnant patients with lung cancer and those requiring ECMO during pregnancy, more extensive and in-depth research are imperative.

## Acknowledgments

We would like to thank the patient who contributed to this study. The project was not funded by any organization.

## Author contributions

**Formal analysis:** Peng Guo, Lilan Wang.

**Writing – original draft:** Peng Guo.

**Writing – review & editing:** Wulan Cao.

## Supplementary Material


