# Supplementary material for: That imaging is necessary to avoid missed diagnoses even in pregnant women: Case report
Source: Medicine (Baltimore). 2025 Aug 8;104(32):e42636. doi: 10.1097/MD.0000000000042636 (PMC12338263; doi:10.1097/MD.0000000000042636)
Supplement: Supplementary file 1 [file medi-104-e42636-s001.docx]

**Supplement 1. Test data of patient at each time point after admission**

| Date  Test | 24 January  2024 | 25 January  2024 | 27 January  2024 | 1 February  2024 | 6 February  2024 | 9 February  2024 |
| --- | --- | --- | --- | --- | --- | --- |
| WBC(10^9^/L) | 13.12 | 18.18 | 20.58 | 12.79 | 7.68 | 8.38 |
| NEUT(%) | 81.8 | 88.7 | 90 | 84.3 | 74 | 72.5 |
| HGB(g/L) | 111 | 88 | 80 | 87 | 84 | 88 |
| PLT(10^9^/L) | 98 | 34 | 37 | 88 | 137 | 320 |
| PCT(ng/ml) | 0.95 | 4.58 | 3.88 | 0.32 | 0.17 | 0.07 |
| P/F(mmHg) | 192 | 305 | 228 | 265 | 320 | 315 |
| Lac(mmol/L) | 4.3 | 10.7 | 1.8 | 1.6 | 0.8 | 0.5 |
| PT(s) | 16.1 | 27.4 | 25.3 | 19.4 | 16.2 | 13.1 |
| APTT(S) | 36 | 53 | 61.6 | 55.8 | 47.7 | 30.9 |
| FIB(g/L) | 1.05 | 0.41 | 1.09 | 0.79 | 1.66 | 3.16 |
| D-Dimer(mg/L) | 2.19 | 134.12 | 60.59 | 125.91 | 62.22 | 4.1 |
| NT-proBNP(pg/ml) | 7126 | 4286 | 5625 |  | 4960 | 4331 |
| ALT(U/L) | 85 | 632 | 564 | 137 | 31 | 18 |
| AST(U/L) | 92 | 2098 | 678 | 49 | 27 | 18 |

WBC(10^9^/L): White Blood Cell; NEUT(%): Neutrophils; HGB(g/L): Hemoglobin; PLT(10^9^/L): platelets; PCT(ng/ml): Procalcitonin; P/F(mmHg): PaO_2_/FiO_2_; Lac(mmol/L): Lactic Acid; PT(s): Prothrombin Time; APTT(S): Activated Partial Thromboplastin Time; FIB(g/L): Fibrinogen; NT-proBNP(pg/ml): N-terminal pro brain natriuretic peptide; ALT(U/L): Alanine Aminotransferase; AST(U/L): Aspartate Aminotransferase.
