# Supplementary material for: That imaging is necessary to avoid missed diagnoses even in pregnant women: Case report
Source: Medicine (Baltimore). 2025 Aug 8;104(32):e42636. doi: 10.1097/MD.0000000000042636 (PMC12338263; doi:10.1097/MD.0000000000042636)
Supplement: Supplementary file 2 [file medi-104-e42636-s002.docx]

Initial echocardiography was performed at 01:52 on January 25, 2024, revealing the following findings:

Right ventricle (RV): 32 mm (normal range: 10-25 mm) ↑

Right atrium (RA) dimensions:

Lateral dimension: 56 mm (normal: 23-45 mm) ↑

Longitudinal dimension: 58 mm (normal: 30-50 mm) ↑

Heart rate (HR): 123 bpm (normal: 60-100 bpm) ↑

Ejection fraction (EF): 74% (normal: 50-85%) ↑

Tricuspid valve assessment:

No leaflet thickening

Color Doppler flow imaging (CDFI): Severe regurgitation (regurgitant area: 12 cm²)

Peak velocity (Vmax): 4.0 m/s

Pressure gradient (PG): 66 mmHg

Pulmonary artery systolic pressure (PASP): 81 mmHg

Echocardiographic Findings:

Right atrial and ventricular dilation

Aortic valve insufficiency (mild)

Tricuspid valve insufficiency (severe)

Pulmonary hypertension (severe)

Pericardial effusion (moderate)

Preserved left ventricular systolic function
